# The effect of representational preference on second language lexical access in late bilinguals

**DOI:** 10.3389/fpsyg.2026.1744494

**Published:** 2026-02-27

**Authors:** Yan Yang, Yi Chang, Qiaozhi Wen, Qinghong Xu

**Affiliations:** 1 School of Foreign Languages, Yan’an University, Yan’an, China; 2 School of Foreign Languages, Yulin University, Yulin, China

**Keywords:** concept-mediation, L1- to concept-mediation conversion, L1-mediation, representational preference, second language vocabulary access

## Abstract

L2 vocabulary learning of late bilinguals is characterized by the mediation of their L1, which may lead to differences in access efficiency and activation pathway among learners with different representational preferences. In the experiment, we used statistical methods to compare the importance of representational preferences with the well-investigated factors, i.e., L2 proficiency and cognitive style, on late bilinguals’ L2 lexical access. The results showed that representational preference was a more effective variable for subject classification. Furthermore, participants with different representational preferences were compared in response time to word translation judgment tasks. The results showed that participants with different representational preferences showed differences in lexical access efficiency, and those with imagistic preference also implied shifts in the access pathway between unfamiliar and familiar words.

## 1 Introduction

Lexical acquisition refers to the process by which repeated exposure enables learners to establish form-meaning connections for novel words within their mental lexicon, thereby facilitating efficient retrieval from memory (Perfetti and Hart, 2002; Bolger et al., 2008; Frishkoff et al., 2008; Schmitt, 2008). A significant proportion of challenges encountered in second language acquisition (SLA) can be attributed to lexical deficits. Suboptimal lexical acquisition further impedes learners’ reading comprehension at the sentence and discourse levels.

### 1.1 Current models of bilingual lexical access and their empirical challenges

The Revised Hierarchical Model (RHM) (Kroll and Stewart, 1994), focusing on language production, posits that late bilinguals possess distinct lexical-form representation systems for their first language (L1) and second language (L2). Crucially, it emphasizes differential strengths in the connections between these lexical forms and the conceptual system, with L1-conceptual links being stronger than L2-conceptual links. Furthermore, the model asserts stronger lexical-level (word-form to word-form) connections from L1 to L2 than from L2 to L1. Conversely, the Bilingual Interactive Activation (BIA/BIA+) model (Dijkstra and van Heuven, 2002), derived from L1 lexical acquisition models and centered on visual word recognition, proposes that L1 and L2 share a single integrated mental lexicon, with lexical recognition achieved through language-non-specific competitive activation and inhibition mechanisms (Kroll et al., 2010).

### 1.2 Discrepant findings and limitations of the RHM model

While influential, the RHM’s predictions have not been uniformly supported. The model posits a developmental shift from lexical mediation (L2→L1 translation via L1 lexical links) to conceptual mediation (direct L2-concept access) with increasing L2 proficiency. However, empirical studies report conflicting patterns. Some support the predicted asymmetry in early learners (e.g., faster L2→L1 translation; Kroll and Stewart, 1994; Duyck and de Houwer, 2008; Duyck and Warlop, 2009), while others find faster L1→L2 translation even for novel or low-proficiency words (e.g., Brysbaert et al., 2014; Hu, 2020), or a lack of asymmetry altogether (e.g., Tseng et al., 2018; Mohsen and Almudawis, 2021; Rice and Tokowicz, 2020). A key limitation is that the RHM does not formally account for lexical competition processes during access. More importantly, it treats bilinguals as a relatively homogeneous group, classifying them primarily by global L2 proficiency. This overlooks stable, underlying individual differences in cognitive processing that may systematically influence how learners rely on lexical versus conceptual pathways, potentially explaining these discrepant results. For instance, individuals with a strong verbal representational preference might develop and rely on direct L2-concept verbal associations differently than those with an imagistic preference, even at similar proficiency levels.

### 1.3 Discrepant findings and limitations of the BIA model

The BIA/BIA+ model, while robust in accounting for cross-linguistic competition in recognition, faces its own empirical challenges when applied to the late bilingual population central to this study. The model’s architecture, emphasizing integrated lexicons and language-non-specific access, does not readily account for the asymmetry in bidirectional lexical activation (i.e., L1-to-L2 vs. L2-to-L1 strength) characteristic of late (or non-balanced) bilinguals (Wei, 2013). Furthermore, it does not explicitly address developmental changes in how L2 lexical forms activate conceptual representations (Yang and Wang, 2023), a core concern in L2 learning research. Studies on conceptual processing often yield mixed findings; some show evidence for shared conceptual stores from early stages (e.g., Duyck and de Houwer, 2008), while others suggest qualitative differences in conceptual activation between L1 and L2 (e.g., Tseng et al., 2018). These discrepancies may arise because the model, in its standard form, does not incorporate individual variation in the quality or modality of conceptual representations (e.g., the relative strength of verbal vs. imagistic conceptual codes), which could moderate the efficiency and pathway of conceptual access for L2 words.

### 1.4 Integrating individual differences: the role of representational preference

The contradictory findings within both theoretical frameworks suggest a critical missing variable: systematic individual differences in cognitive representation. The Dual Coding Theory (DCT) (Paivio, 1980) posits two functionally independent but interconnected systems for information representation: a verbal system and a non-verbal (imagistic) system. Furthermore, Hayes et al. (2020) demonstrated that during the information acquisition phase, regardless of whether words or images of the objects they denote are presented, verbal-preferential individuals consistently represent information verbally, while imagistic-preferential individuals favor imagistic representation. This underscores the stability of representational preference in information processing. Individuals exhibit stable representational preferences, tending to favor one modality over the other during encoding and retrieval (Riding et al., 2011; Sun, 2018; Kang et al., 2013).

Among late bilinguals, the majority utilize both imagistic and verbal modalities for representation, classifying them as dual-preferential users (Yao et al., 2010; Alfred et al., 2020). However, approximately 35% of all individuals prefer a single representational modality, either imagistic or verbal (Sanjanaashree et al., 2014). Research involving native Chinese speakers indicates that an individual’s representational preference influences the effectiveness of their L1 abstract word memorization. Specifically, significant differences in explicit memory performance for L1 abstract words exist between imagistic-preferential and verbal-preferential individuals. This discrepancy arises because imagistic-preferential individuals process abstract words, which rely more heavily on verbal representation, less efficiently than their verbal-preferential counterparts (Niu and Zhao, 2005).

For late bilinguals, whose L2 lexical-conceptual links are initially weak, this preference may fundamentally shape the early mediation pathways. An imagistic-preferential learner, when acquiring a new L2 word, might be compelled to rely on L1 lexical mediation to first access a conceptual image, whereas a verbal-preferential learner could establish a more direct L2 verbal-conceptual link. This aligns with the Encoding-Retrieval Compatibility principle (Eysenck and Keane, 2010; Xiao et al., 2020; Kang et al., 2013), suggesting these differential encoding pathways will recur during retrieval, affecting lexical access efficiency. Therefore, representational preference is not merely a supplementary factor; it is a candidate cognitive construct that can elucidate why the predictions of RHM and BIA show such variability across studies and participants. By explicitly modeling this preference, we can move beyond classifying bilinguals solely by proficiency and begin to explain divergent access patterns within proficiency groups.

Currently, L2 lexical access research has rarely accounted for representational preference distinctions among participants, indicating that existing studies have largely overlooked the influence of individual cognitive traits on L2 lexical access. Due to the absence of direct links between the L2 lexical system and the conceptual system in late bilinguals, coupled with the weaker associative networks within the L2 lexical system itself, imagistic-preferential individuals might be compelled, particularly during initial L2 learning or the acquisition of novel L2 words, to initially rely on mediation through the L1 lexical system to activate the imagistic system for conceptual access subsequently. In contrast, verbal-preferential individuals can complete this process without activating the imagistic system. The Revised Hierarchical Model (RHM) proposes that the connection between L2 lexical form and concept develops from a “lexical mediation” stage via L1 to a “conceptual mediation” stage as L2 proficiency increases. Consequently, the lexical access and retrieval differences between late bilinguals with different representational preferences should be more pronounced for L2 than L1 vocabulary.

For imagistic-preferential individuals, the mediation pathway via the L1 lexical system conflicts with their representational preference. Therefore, the semantic access pathway for L2 words may undergo a developmental shift: from reliance on the L1 lexical system pathway when processing unfamiliar words, toward an imagistic system pathway analogous to L1 lexical access when processing familiar words. However, unbalanced verbal-preferential bilinguals, being adept at L1 lexical representation, do not necessitate this “pathway shift.” For dual-preferential users, overcoming competition between the two representational modalities is consistent throughout the representation of unfamiliar and familiar L1 words.

Representational preference constitutes a significant individual cognitive factor influencing the process of L2 lexical access, as evidenced in related research. To examine the hypotheses above and integrate the factor of individual representational preference into existing lexical access theories—thereby advancing the understanding of the cognitive mechanisms underlying lexical learning—this study conducts an experiment that employs statistical methods with a lexical translation judgment task, aiming to investigate whether classifying participants based on representational preference constitutes a more effective cognitive classification variable for understanding L2 semantic access, compared to the conventionally used variables, such as cognitive style and L2 proficiency, etc. in prior research (Hu, 2020). Then it investigates potential differences in task performance across L2reference constitutes a more effective cognitive classification variable for understanding L2 semantic access, istic, dual), examining whether the three participant groups exhibit fundamentally distinct lexical access patterns.

## 2 Methodology

### 2.1 Participants

A priori power analysis was conducted using G*Power 3.1 (Faul et al., 2009) for a between-subjects ANOVA (three groups) with an alpha of.05, power of.80, and a medium effect size (*f* = 0.25) (Cohen, 1992). The analysis indicated a required total sample size of 78 participants (i.e., 26 participants per group). To ensure robust group comparisons and account for potential data loss, we recruited a larger sample of 160 participants. The 160 participants were undergraduates (59 males), aged 19–25 years (*M* = 21.13, SD = 2.46), with Mandarin as their first language and approximately 10 years of English learning experience (*M* = 8.71, SD = 1.60). All participants volunteered for the experiment, completed questionnaires, and received monetary compensation upon completion. Following the assessment of representational preference (see section 2.3), 35 participants were randomly selected from each of the three preference groups (verbal, imagistic, dual) for the subsequent translation judgment task, meeting and exceeding the power requirement.

### 2.2 Measurement of control variables

Cognitive style: Cognitive style was assessed using the Santa Barbara Learning Style Questionnaire (SBCSQ) (Mayer and Massa, 2003). This self-report instrument consists of six scenario-based questions where participants indicate their preference for verbal or visual information processing on a 7-point Likert scale.

Verbal working memory capacity: Verbal Working Memory Capacity was measured using the Digit Span subtest from the Wechsler Adult Intelligence Scale-Revised in China (WAIS-RC). Both the Forward and Backward recall tasks were administered, and the score was used as the index of working memory capacity.

Vocabulary size: An online assessment tool^1^ was used to estimate participants’ receptive English vocabulary size (Fokin et al., 2025; Keuleers et al., 2015; Stoeckel et al., 2019).

Household income level: A 7-point scale (lowest-income households, low-income households, lower-middle-income households, middle-income households, upper-middle-income households, high-income households, highest-income households) based on the household income classification system used in the China Family Panel Studies (i.e., CFPS)^2^ was administered to measure participants’ family economic status.

Subjective socioeconomic status (SES): The Subjective SES Scale (Adler et al., 2000) was used to measure participants’ perceived family economic standing.

L2 experience and aptitude: The Language History Questionnaire 2.0 Simplified Chinese version (LHQ 2.0; Li et al., 2014)^3^ was administered to assess L2 experience and self-reported learning aptitude.

Objective L2 proficiency: Participants reported their most recent College English Test Band 4 (CET-4) score as an objective measure of L2 proficiency.

Subjective L2 proficiency: Participants rated their overall L2 proficiency on a scale from 1 (not proficient) to 10 (highly proficient).

Complete description and coding scheme of Control Variables are listed in Table 1.

**TABLE 1:** Variable coding scheme.

Variable	Coding
Preferential modality	1: dual; 2: verbal; 3: imagistic
Cognitive style	1: verbal; 2: visual
Subjective L2 proficiency	1–10 (low → high)
Age	1: 19; 2: 20; 3: 21; 4: 22; 5: 23; 6: 24; 7: 25
Gender	1: male; 2: female
Language aptitude	1–7 (low → high)
Objective L2 proficiency	1–7 (corresponding to CET-4 percentiles)
L2 learning experience	1: 10 years; 2: 11 years; 3: 12 years; 4: 13 years; 5: 14 years; 6: 15 years; 7: ≥ 16 years
L2 usage frequency	1–7 (daily average hours: 1 = < 1 h; 7 ≥ 6 h)
Household income	1: < 20 k; 2: 30–40 k; 3: 50–60 k; 4: 70–80 k; 5: 90–100 k; 6: 110–240 k; 7: > 250 k (CNY)
SES	1–10 (low → high; societal percentile)
L2 vocabulary size	1–7 (per 1,000-word interval)
Working memory	1–22 (raw score; low → high)

### 2.3 Measurement of representational preference

Stimuli: Adopting the Verbal Attentional Task (VAT) paradigm from Alfred et al. (2020), word-picture paired stimuli were created. The pictorial stimuli consisted of spade, club, and diamond symbols. Three congruent word-picture pairs (e.g., “黑桃” [spade] + ♠◻) and six incongruent pairs (e.g., “梅花” [club] + ♦◻) were designed. Vertical positions (word above/below image) were counterbalanced, resulting in six congruent and twelve incongruent stimulus pairs (see Table 2).

**TABLE 2:** Sample stimuli for preferential modality measurement.

Stimulus type	Example
Word-image congruent	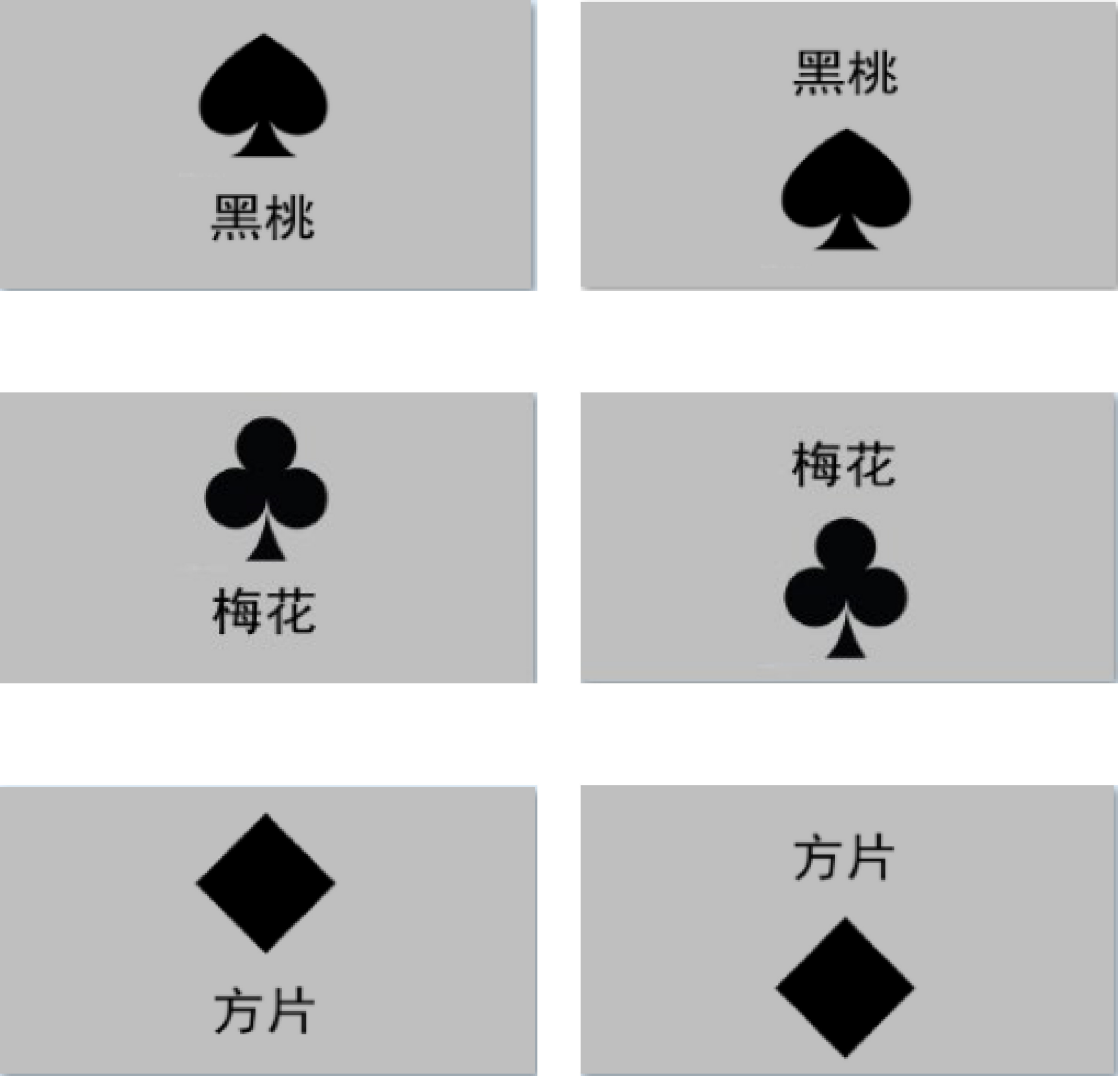
Word-image incongruent	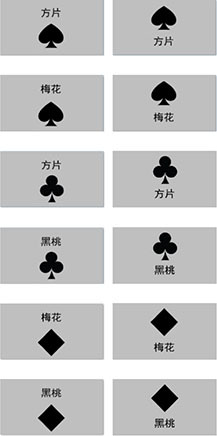

Procedure: Stimuli were presented via E-Prime 2.0. Each trial began with a central fixation cross (“+”) for 2000 ms, followed by an 80-ms presentation of the stimulus pair. A blank screen then appeared for 2000 ms, during which participants pressed “J” for spade, “K” for club, and “L” for diamond on the keyboard. Trials advanced automatically after a keypress or a timeout (2000 ms).

The formal experiment included 144 congruent trials (75%) and 48 incongruent trials (25%), totaling 192 trials (Alfred et al., 2020; Hayes et al., 2020). Practice trials (congruent-only; 20 trials) required > 80% accuracy to proceed to the formal experiment. Participants were not informed about incongruent trials.

Scoring: The VAT score was calculated based on the 48 incongruent trials only, as these trials force a choice between verbal and imagistic information. For each incongruent trial, the response could be congruent with the word or with the picture. The score was calculated using the formula: (Number of word-congruent responses - Number of image-congruent responses)/Total valid incongruent trials. This calculation yields a score ranging from −1 to +1. Scores approaching +1 indicate a strong preference for verbal representation, while scores approaching −1 indicate a strong preference for imagistic representation, and scores near 0 indicate no strong preference (dual-preference).

**FIGURE 1:**
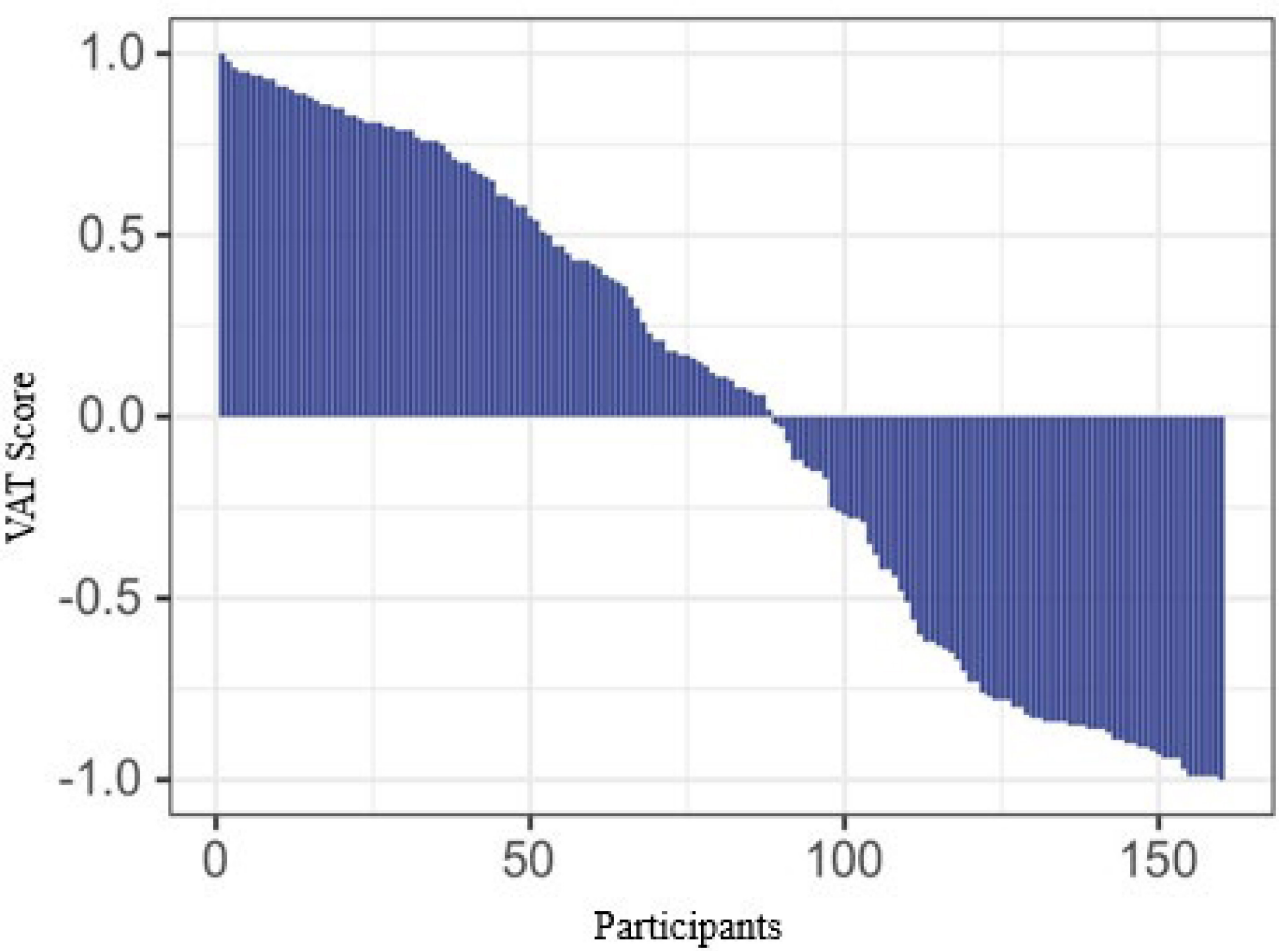
VAT score distribution of 160 participants.

Measurement outcomes of representational preference are presented in Figure 1. Participants were then grouped according to established VAT score thresholds, i.e., Verbal-preferential group (VAT scores ≥ +0.75; *n* = 35); Imagistic-preferential group (VAT scores ≤ −0.75; *n* = 39); Dual-preferential group (*n* = 86). For subsequent experimental procedures, the Dual-preferential group was further refined to include only those with VAT scores between -0.4 and +0.4 (as per Alfred et al., 2020; Hayes et al., 2020).

### 2.4 L2→L1 and L1→L2 translation judgment task

Material: High- and low-frequency L2 lexical stimuli were selected from the Snodgrass and Vanderwart standardized picture set (Snodgrass and Yuditsky, 1996). Before the main experiment, 20 university L2 English learners (who did not participate in the main experiment) rated the familiarity and complexity of the pictures and their corresponding English words on 7-point scales (1 = completely unfamiliar, 7 = highly familiar; 1 = extremely low complexity, 7 = extremely high complexity). Based on these ratings, 10 English words with familiarity ratings ≥ 6.5 were selected as Familiar Words; 10 English words with familiarity ratings between 2.3 and 4.5 were selected as Novel Words (Li et al., 2011). The complete stimulus set was created with corresponding pictures (complete materials in Supplementary Appendix).

**FIGURE 2:**
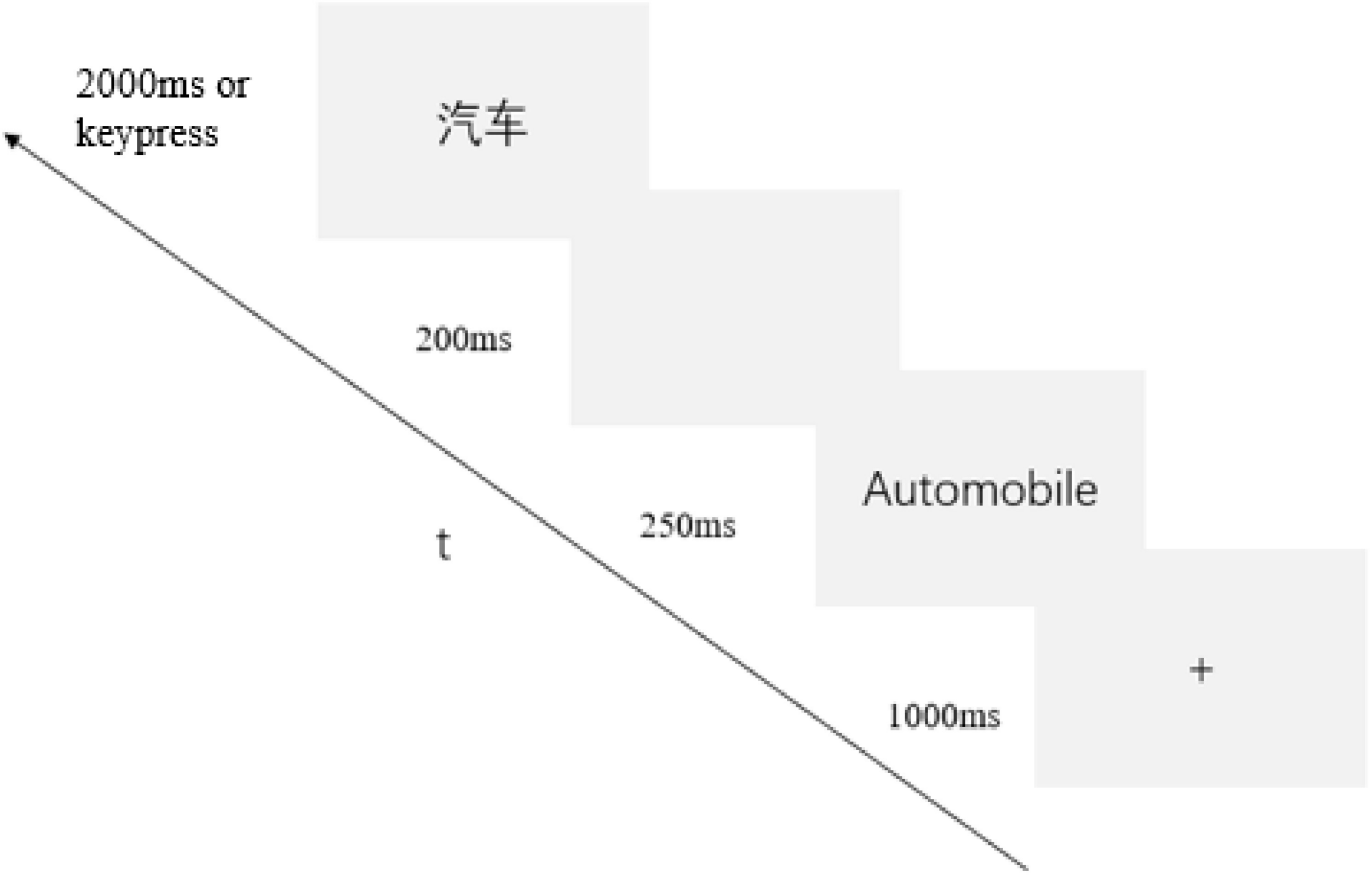
Procedure for the test phase.

**FIGURE 3:**
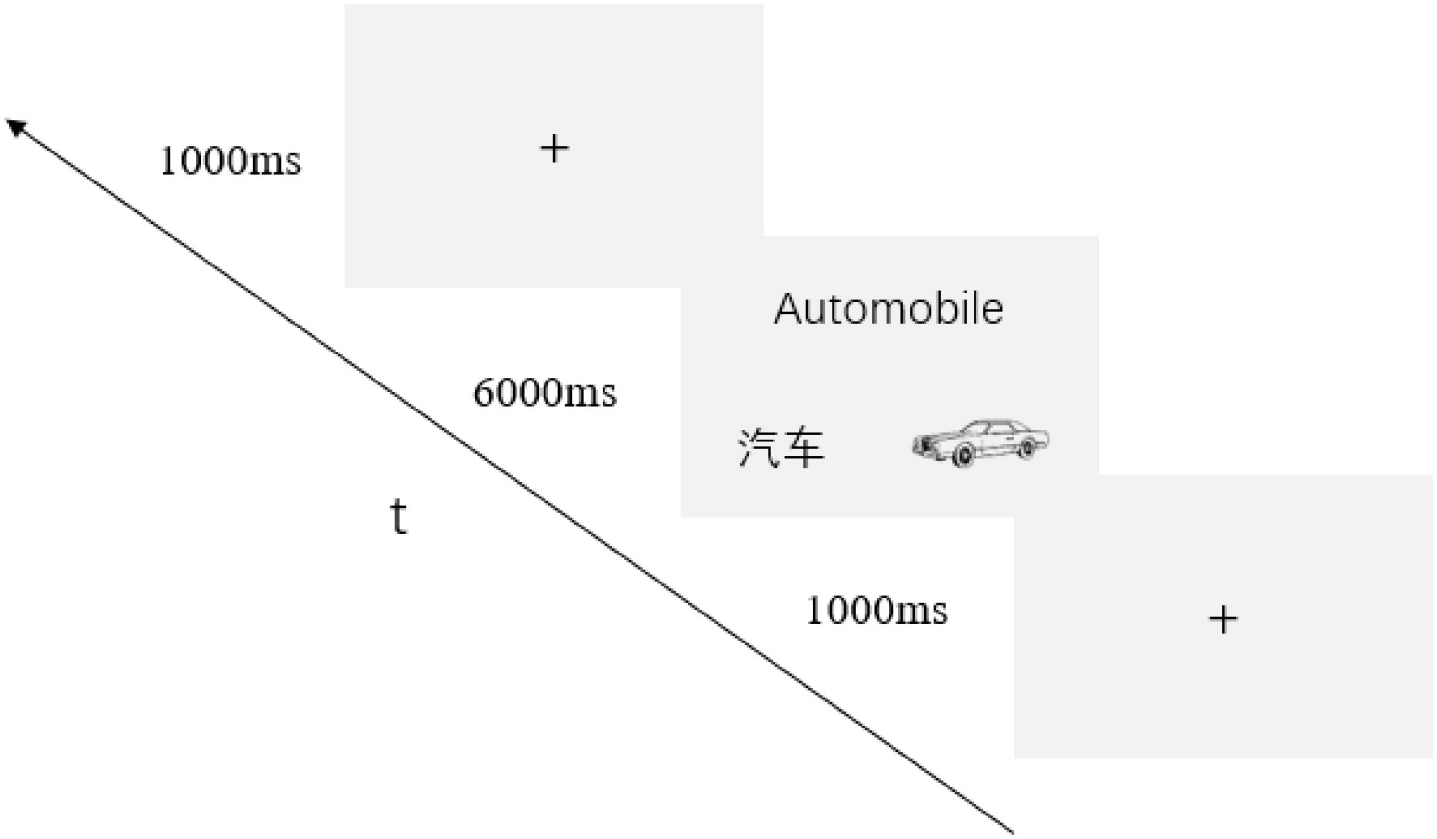
Procedure for the learning phase.

Procedure: Stimuli were presented using E-Prime 2.0. The experimental process for novel words involved: (1) A learning phase (2 repetitions) with 1,000 ms fixation followed by 6,000 ms simultaneous presentation of English words, Chinese translations, and pictures (Bartolotti and Marian, 2014; Bartolotti and Marian, 2017; Bai et al., 2011); (2) A 5-min serial subtraction by three interference task (Kole and Healy, 2013; Dikmans et al., 2020); (3) A test phase where 1,000 ms fixation preceded L2 English words, followed by 200 ms blanks and translation prompts requiring speeded correct/incorrect judgments (disappearing upon response or after 2,000 ms; see Figure 2). Familiar words tasks appeared only in the test phase without prior notification of familiarity status. Each word generated correct/incorrect trials (incorrect trials used random lexical distractors from the material words), presented 3 times in randomized order (60 trials per direction; 120 trials in total), preceded by 20 practice trials (see Figure 3).

### 2.5 Data processing

Data was processed and analyzed using SPSS 25.0. Data processing entailed the exclusion of 1 verbal-preferential participant whose accuracy fell below 75% across familiar word sequences, alongside 23 participants (8 dual-preferential, 8 imagistic-preferential, 7 verbal-preferential) failing to meet the 75% accuracy threshold in novel word sequences. Reaction time (RT) data from correct trials were subsequently analyzed, with outliers exceeding ± 2 standard deviations removed; these extreme values constituted less than 5% of the dataset.

Standardized residual histograms and normal P-P plots were generated for RT data across four task conditions: novel words (L2→L1; L1→L2) and familiar words (L2→L1; L1→L2). Each to verify normality of error distributions. Residual independence was assessed via Durbin-Watson tests, yielding values of 1.319 (novel L2→L1), 1.091 (novel L1→L2), 2.053 (familiar L2→L1), and 1.887 (familiar L1→L2). Multiple linear regression analyses were then conducted for each task condition, with representational preference (dual/imagistic/verbal), L2 proficiency, and cognitive style (visual/verbal) as predictors. The significance of regression coefficients (evaluated via *t*-tests) determined predictive differences among these independent variables on task-specific RTs (Yuan, 2019). Control variables included age, gender, language aptitude, L2 learning experience, objective English proficiency, usage frequency, subjective socioeconomic status, household income, working memory, and vocabulary size. Following multicollinearity diagnostics using Variance Inflation Factors (VIF) and Tolerance indices, two variables exhibiting high collinearity were removed: L2 learning experience (VIF = 8.016, Tolerance = 0.125) and subjective socioeconomic status (VIF = 8.483, Tolerance = 0.118). All retained variables demonstrated VIF < 2 and Tolerance > 0.5, confirming acceptable multicollinearity levels (Conversano et al., 2020).

To test our main hypotheses, we conducted: (1) Multiple linear regressions for each translation direction (L1→L2, L2→L1) and word type (familiar, novel) with representational preference, L2 proficiency, and cognitive style as predictors; (2) Separate mixed-design ANOVAs on RTs for familiar and novel words, with Representational Preference (three groups) as a between-subjects factor and Translation Direction (L1→L2, L2→L1) as a within-subjects factor.

## 3 Results

Results indicated that both representational preference and L2 proficiency demonstrated significant regression coefficients across translation directions for familiar word sequences. However, the coefficient significance for representational preference (*t* = −3.567, *p* = 0.001 for L1→L2; *t* = −4.264, *p* < 0.001 for L2→L1) exceeded that of L2 proficiency (*t* = 1.972, *p* = 0.052 for L1→L2; *t* = 2.110, *p* = 0.038 for L2→L1). Cognitive style showed non-significant effects in both directions (*p* = 0.452 for L1→L2; *p* = 0.625 for L2→L1; see Table 3). For novel word sequences, neither representational preference (*p* = 0.240 for L1→L2; *p* = 0.066 for L2→L1), cognitive style (*p* = 0.577 for L1→L2; *p* = 0.314 for L2→L1), nor L2 proficiency (*p* = 0.973 for L1→L2; *p* = 0.591 for L2→L1) reached significance. Notably, representational preference approached significance in the L2→L1 condition (*t* = −1.860, *p* = 0.066; see Table 4).

**TABLE 3:** Multiple linear regression of three predictors on reaction times for familiar words by translation direction.

	Direction	β	*SE*	*t*	*p*
Preferential modality	L1-L2	-45.493	12.752	-3.567	0.001
	L2-L1	-50.057	11.738	-4.264	0.000
Cognitive style	L1-L2	16.582	21.969	0.755	0.452
	L2-L1	9.918	20.223	0.490	0.625
L2 proficiency	L1-L2	43.253	21.933	1.972	0.052
	L2-L1	42.593	20.190	2.110	0.038

**TABLE 4:** Multiple linear regression of three predictors on reaction times for novel words by translation direction.

	Direction	β	*SE*	*t*	*p*
Preferential modality	L1-L2	-33.783	28.568	-1.183	0.240
	L2-L1	-52.403	28.171	-1.860	0.066
Cognitive style	L1-L2	26.187	46.753	0.560	0.577
	L2-L1	46.721	46.102	1.103	0.314
L2 proficiency	L1-L2	-1.587	47.239	-0.034	0.973
	L2-L1	25.140	46.582	0.540	0.591

Results revealed that representational preference demonstrated a more robust relationship with L2 lexical access efficiency than cognitive style and L2 proficiency (variables conventionally emphasized in L2 lexical processing research). This suggests that representational preference may constitute a more effective cognitive between-subjects variable. Although superficially analogous, representational preference and cognitive style are fundamentally distinct constructs. First, representational preference is assessed via behavioral tasks (VAT tasks; Alfred et al., 2020), whereas cognitive style is typically measured through self-report questionnaires (directly or scenario-based inquiries about verbal/imagistic preferences; Mayer and Massa, 2003). Second, extant research (Alfred et al., 2020; Hayes et al., 2020) indicates that cognitive style fails to accurately reflect neural activation patterns during information processing, with imagistic/verbal representational preferences not mapping directly onto verbal/visual cognitive style dimensions. Finally, studies show inconsistent outcomes across cognitive style questionnaires, raising concerns about their psychometric reliability (Mayer and Massa, 2003). In essence, methodological and empirical evidence confirms divergent outcomes: behavioral measures of representational preference capture real-time processing effects through reaction times, whereas questionnaire-based cognitive style assessments lack such sensitivity.

Critically, results demonstrated that representational preference exerted more potent effects than cognitive style and L2 proficiency in the translation judgment task, positioning it as a distinct and valid cognitive variable. Hence, the present study further investigates: (1) whether differences in representational preference among late bilinguals impact L2 lexical access, and (2) whether the access pathways align with predictions of the Revised Hierarchical Model (RHM). Thus, we further employed a 3 (representational preference: imagistic, verbal, dual) × 2 (task direction: L2→L1, L1→L2) mixed factorial analysis on RTs of L2→L1 and L1→L2 Translation Judgment Task, with representational preference as a between-subjects factor and task direction as a within-subjects factor. The analysis was implemented across two experimental blocks: familiar words and novel words. Preliminary analyses confirmed no statistically significant between-group differences on control variables (see Table 5).

**TABLE 5:** Between-group comparisons of control variables across preferential modality groups (M ± SD).

Variable	Imagistic (*n* = 27)	Dual (*n* = 27)	Verbal (*n* = 27)	*F*(2, 80)	*p*
L2 proficiency	4.81 (1.30)	5.03 (1.26)	4.81 (1.08)	0.32	0.73
Language aptitude	4.22 (1.78)	3.69 (1.77)	3.85 (1.83)	0.67	0.52
L2 Usage	1.12 (0.79)	1.02 (0.77)	1.30 (0.80)	0.94	0.40
L2 Vocabulary size	4.35 (1.47)	4.18 (1.59)	4.53 (1.65)	0.37	0.69
Working memory	15.63 (2.15)	15.56 (2.47)	15.52 (2.95)	0.02	0.98
CET-4 score	480.52 (46.98)	477.84 (40.49)	476.74 (55.22)	0.05	0.96
Household income	4.07 (1.33)	3.66 (1.33)	3.89 (0.93)	0.87	0.42
SES	4.70 (1.59)	4.31 (1.45)	4.33 (1.47)	0.60	0.55
L2 Learning experience	4.74 (1.75)	4.63 (1.52)	4.74 (1.56)	0.05	0.95

**FIGURE 4:**
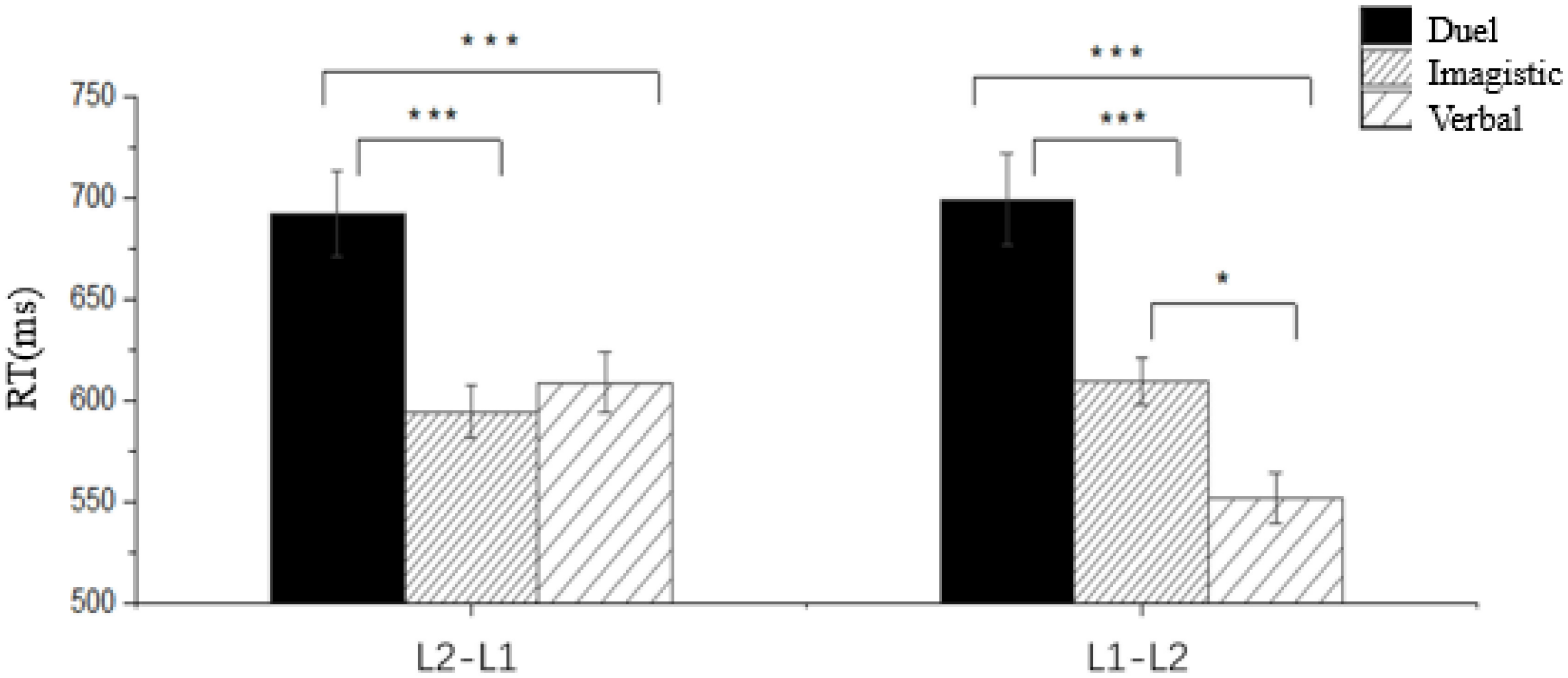
Reaction times for familiar word translation tasks across preferential modality groups and directions. Error bars represent standard error of the mean. *p < 0.05, ***p < 0.001.

Repeated-measures ANOVA on reaction times for the familiar word sequence revealed a significant main effect of representational preference [*F*(2, 156) = 30.913, *p* < .001, η^2^ = 0.284], with Bonferroni-corrected *post-hoc* tests indicating significantly shorter reaction times for both imagistic-preferential (*p* < 0.001) and verbal-preferential groups (*p* < 0.001) relative to the dual-preferential group. However, no significant difference emerged between imagistic and verbal groups (*p* = 0.342). A significant interaction between representational preference and task direction was observed [*F*(2, 156) = 3.966, *p* = 0.021, η^2^ = 0.048]. Simple effects analysis demonstrated that under the L2→L1 condition, significant group differences existed [*F*(2, 156) = 11.128, *p* < 0.001, η^2^ = 0.125], where dual-preferential participants exhibited longer reaction times than both imagistic (*p* < 0.001) and verbal groups (*p* = 0.001), while imagistic-verbal differences remained non-significant. Conversely, for the L1 longer reaction times than both differences were found [*F*(2, 156) = 24.081, *p* < 0.001, η^2^ = 0.236], with verbal-preferential participants responding faster than imagistic (*p* = 0.01) and dual groups (*p* < 0.001), and imagistic-preferential faster than dual-preferential (*p* < 0.001). Paired-samples *t*-tests within groups further revealed that imagistic-preferential participants showed shorter reaction times for L2→L1 versus L1→L2 (*p* = 0.021, Cohen’s *d* = 0.204), whereas verbal-preferential participants demonstrated the reverse pattern with shorter reaction times for L1→L2 versus L2→L1 (*p* < 0.001, Cohen’s *d* = 0.831; see Figure 4), and dual-preferential participants exhibited no significant directional difference (*p* = 0.541).

**FIGURE 5:**
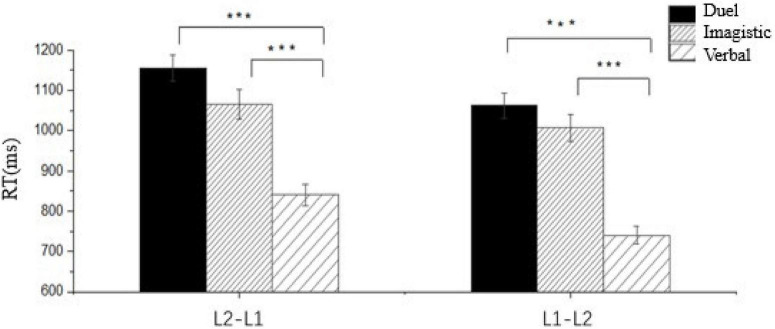
Reaction times for novel word translation tasks across preferential modality groups and directions. Error bars represent standard error of the mean. ***p < 0.001.

Repeated-measures ANOVA on reaction times for the novel word sequence revealed a significant main effect of representational preference [*F*(2, 156) = 57.833, *p* < 0.001, η^2^ = 0.411], with *post-hoc* analyses indicating significantly shorter reaction times for verbal-preferential participants compared to imagistic (*p* < 0.001) and dual-preferential groups (*p* < 0.001), and marginally shorter reaction times for imagistic-preferential relative to dual-preferential participants (*p* = 0.053). A significant main effect of task direction emerged [*F*(1, 156) = 11.038, *p* = 0.001, η^2^ = 0.062], with faster responses for L1→L2 than L2→L1 translations. Paired-samples *t*-tests within each group confirmed significantly shorter reaction times for L1→L2 versus L2→L1 across all representational preferences: dual-preferential (*p* = 0.003, Cohen’s *d* = 0.533), imagistic-preferential (*p* = 0.042, Cohen’s *d* = 0.320), and verbal-preferential (*p* < 0.001, Cohen’s *d* = 0.790; see Figure 5).

## 4 Discussion

The Revised Hierarchical Model (RHM) asserts translation asymmetry in late bilinguals, reflecting differential reliance on lexical form versus conceptual activation (Kroll and Stewart, 1994, p. 168). While RHM predicts faster L2→L1 translations due to stronger lexical links, the novel word findings contradict this: all groups showed faster L1→L2 responses (see Table 6). This likely stems from shared L1-L2 conceptual networks (van Heuven and Dijkstra, 2010; Zeng et al., 2020), where L1→L2 tasks efficiently activate concepts via the dominant L1 verbal system. For familiar words, however, comparable L1→L2 and L2→L1 reaction times suggest near-native conceptual access efficiency (see Table 7).

**TABLE 6:** Reaction times (ms) for novel word translation tasks by direction and preferential modality group (M ± SD).

Direction	Duel	Imagistic	Verbal
L2-L1	1156.18 (176.47)	1065.24 (187.95)	840.59 (135.78)
L1-L2	1062.18 (180.21)	1006.63 (177.84)	741.04 (115.53)

**TABLE 7:** Reaction times (ms) for familiar word translation tasks by direction and preferential modality group (M ± SD).

Direction	Duel	Imagistic	Verbal
L2-L1	692.15 (128.27)	594.20 (78.89)	608.63 (74.95)
L1-L2	699.20 (136.68)	609.51 (70.76)	551.31 (62.48)

Critically, distinct patterns emerged across representational preferences. For novel words, all groups exhibited faster L1→L2 than L2→L1 responses. For familiar words: verbal-preferential learners maintained faster L1→L2 responses; imagistic-preferential learners reversed to faster L2→L1 responses; dual-preferential learners showed no directional difference. This reversal in imagistic-preferential learners possibly signifies a activation pathway shift (Brysbaert and Duyck, 2010): repeated exposure enables transition from L1-verbal mediation to direct L2-imagery-concept access (their preferred modality), matching verbal learners’ efficiency in L2→L1 tasks. However, L1→L2 tasks still require verbal mediation (L1 wordform → imagistic system → semantic system → L2 wordform), explaining their slower L1→L2 responses versus verbal learners. Dual-preferential learners’ persistently slow RTs possibly indicate unresolved verbal/imagistic competition. These findings also necessitate distinguishing novel/familiar word processing.

Research guided by the Revised Hierarchical Model (RHM) predominantly classifies bilinguals by L2 proficiency, typically measured via subjective self-ratings. However, studies employing high- versus low-proficiency cohorts yield conflicting conclusions (Barcroft, 2009). This approach implicitly assumes inter-group distinctions manifest solely in processing efficiency rather than fundamental differences in access mechanisms. While some investigations explore individual differences through cognitive style taxonomies, questionnaire-derived classifications fail to capture actual information-processing strategies during experimental tasks reliably (Alfred et al., 2020).

Dual Coding Theory (DCT) offers a neurocognitively grounded alternative, proposing two functionally autonomous yet integrated multimodal systems: an internalized nonverbal system encoding perceptual properties of objects/images, and a verbal system specialized for linguistic properties (Paivio, 2010). Neuroimaging evidence reveals striking intra-individual consistency in BOLD-fMRI activation patterns across episodic, working, and semantic memory retrieval tasks, with representational preference robustly predicting task-specific neural signatures (Hsu et al., 2011; Miller et al., 2012). Critically, lifelong engagement of preferred processing modality strengthens connectivity within modality-specific cortical networks (Soares et al., 2013), reinforcing an individual’s representational bias. This neuroplasticity is structurally observable: verbal-preferential individuals exhibit heightened reliance on left peri-sylvian regions (sylvian fissure and supramarginal gyrus) during verbal/imagistic processing, accompanied by volumetric increases in bilateral language-related gray and white matter (Hayes et al., 2020; Alfred et al., 2019).

As lexical access epitomizes information encoding and retrieval, representational preference likely constitutes a fundamental individual difference variable in L2 lexical processing yet remains conspicuously absent in extant bilingual access literature. RHM posits that lexical mediation transitions from L1-dependent to concept-dependent pathways with increasing proficiency, evidenced by distinct prefrontal activation patterns in novice versus proficient bilinguals (Marian et al., 2014; Kroll and Bialystok, 2013). Further corroborating this dichotomy, L1 lexical access preferentially recruits posterior parietal regions, whereas novel L2 words engage the left hippocampus (Bartolotti and Marian, 2017), suggesting distinct cortical circuits for L1 versus novel L2 processing. This implies divergent cognitive architectures underlying novel versus familiar L2 word access.

## 5 Conclusion

Conventional paradigms assume uniform access mechanisms within each word type (novel/familiar) across all participants. Experiment 2 decisively challenges this assumption: novel words elicited uniformly faster L1→L2 responses across groups, contradicting RHM but aligning with shared conceptual network models (van Heuven and Dijkstra, 2010). For familiar words, however, groups exhibited distinct mediational pathways: imagistic-preferential learners demonstrated a shift toward imagery-mediated access, verbal-preferential learners maintained verbal-conceptual mediation, and dual-preferential learners showed persistent cross-system competition. Crucially, neglecting these preference-driven differential pathways introduces a methodological confound: sampling bias interacting with unaccounted word-type processing differences likely explains contradictory RHM validations in prior literature.

Therefore, comprehensive models of lexical access should integrate representational preference as a key variable. This paradigm shift enables more precise characterization of how late bilinguals establish form-meaning connections across developmental stages and lexical categories, ultimately reconciling longstanding theoretical discrepancies.

This study demonstrates that representational preference holds greater significance than conventionally employed factors like L2 proficiency and cognitive style in research on lexical access among late bilinguals. As a systematic individual difference, representational preference relates to both the efficiency and pathways of L2 lexical access. While our overall findings do not support the RHM model, they yield a plausible interpretation through the lens of Dual Coding Theory. These results suggest potential for theoretical integration between these frameworks in advancing our understanding of L2 lexical access mechanisms.

## 6 Limitations

Several limitations of this study should be acknowledged. First, while the use of standardized stimuli ensured internal validity, the limited set of concrete nouns may constrain the generalizability of the findings across diverse semantic categories or parts of speech. Second, the observed reversal of the classic L2→L1 translation advantage for novel words, while interpretable through shared conceptual networks, highlights the need for more nuanced theoretical models that account for task-specific and developmental influences on access pathways. Finally, the present evidence is strictly behavioral; although the construct of representational preference is grounded in neurocognitive literature, direct neural correlates of the proposed pathway shifts remain to be empirically verified. Future research employing a broader range of lexical items, longitudinal designs, and multimodal methods (e.g., neuroimaging) will be essential to validate and extend these findings.

## Data Availability

The raw data supporting the conclusions of this article will be made available by the authors, without undue reservation.

## Footnotes

1
http://testyourvocab.com

2
http://www.isss.pku.edu.cn/cfps

3
http://blclab.org/lhq2/
