# Supplementary material for: The effect of representational preference on second language lexical access in late bilinguals
Source: Front Psychol. 2026 Feb 27;17:1744494. doi: 10.3389/fpsyg.2026.1744494 (PMC12982423; doi:10.3389/fpsyg.2026.1744494)
Supplement: Supplementary file 1 [file Table_1.pdf]

List of Experimental Materials in L2→L1 and L1→L2 Translation Judgment Tasks

	English words	Chinese translation	Picture
	jackass	驴	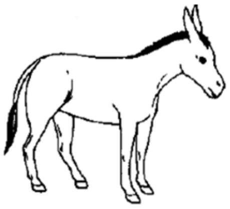
	revolver	手枪	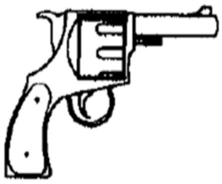
	copter	直升机	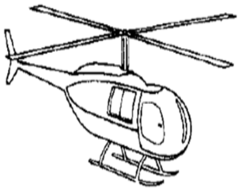
Unfamiliar words	cottage	房子	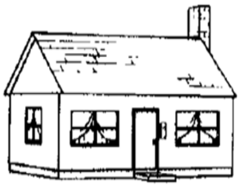
	pullover	毛衣	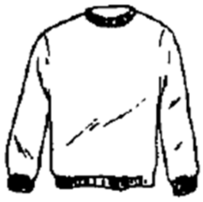
	locomotive	火车	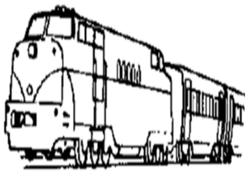

	bugle	小号	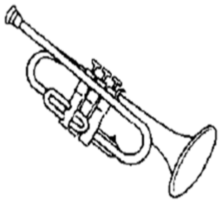
	tortoise	乌龟	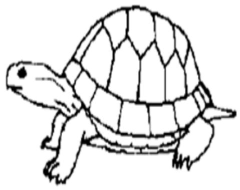
	cello	提琴	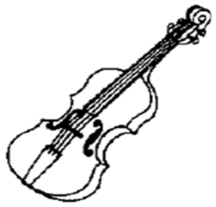
	goblet	杯子	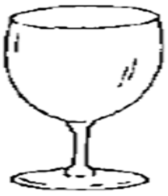
Familiar words	eye	眼睛	-----
	lion	狮子	-----
	pig	猪	-----
	shoe	鞋子	-----
	table	桌子	-----
	book	书本	-----
	car	汽车	-----
	flower	花朵	-----
	tree	大树	-----
	watch	手表	-----
